# The Method of Low-Temperature ICP Etching of InP/InGaAsP Heterostructures in Cl_2_-Based Plasma for Integrated Optics Applications

**DOI:** 10.3390/mi12121535

**Published:** 2021-12-10

**Authors:** Sergey Ishutkin, Vadim Arykov, Igor Yunusov, Mikhail Stepanenko, Vyacheslav Smirnov, Pavel Troyan, Yury Zhidik

**Affiliations:** 1 Micran, Research and Production Company, 634041 Tomsk, Russia; snorlox111@yandex.ru; 2 Integrated Optics and Microwave Photonics Laboratory, Tomsk State University of Control System and Radioelectronics, 634050 Tomsk, Russia; arykov.v@ir-mw.com (V.A.); yunusov.i@ir-mw.com (I.Y.); stepanenko.m@ir-mw.com (M.S.); tpe@tusur.ru (P.T.); zhidikyur@mail.ru (Y.Z.)

**Keywords:** ICP etching, waveguides, InP, cyclic etching, plasma polishing

## Abstract

Chlorine processes are widely used for the formation of waveguide structures in InP-based optoelectronics. Traditionally, ICP etching of InP in a Cl_2_-based plasma requires substrate temperatures in the range of 150–200 °C. This condition is mandatory, since during the etching process low-volatility InCl_x_ components are formed and at insufficient temperatures are deposited onto substrate, leading to the formation of defects and further impossibility of the formation of waveguide structures. The need to preheat the substrate limits the application of chlorine processes. This paper presents a method of ICP etching an InP/InGaAsP heterostructure in a Cl_2_/Ar/N_2_ gas mixture. A feature of the developed method is the cyclic etching of the heterostructure without preliminary heating. The etching process starts at room temperature. In the optimal etching mode, the angle of inclination of the sidewalls of the waveguides reached 88.8° at an etching depth of more than 4.5 μm. At the same time, the surface roughness did not exceed 30 nm. The selectivity of the etching process with respect to the SiN_x_ mask was equal to 9. Using the developed etching method, test integrated waveguide elements were fabricated. The fabricated active integrated waveguide (p-InP epitaxial layers were not removed) with a width of 2 μm demonstrated an optical loss around 11 ± 1.5 dB/cm at 1550 nm. The insertion loss of the developed Y- and MMI-splitters did not exceed 0.8 dB.

## 1. Introduction

At present, integrated optoelectronics continues to be a dynamically evolved area. This is primarily facilitated by the fact that photonic integrated circuits (PICs) are widely used in the construction of telecommunication networks. Continuously growing volumes of data traffic require an infrastructure that is built on a corresponding component base. Traditional electric communication lines have long ceased to satisfy the demand for bandwidth, and not only the backbone data transmission lines; today they are actively being replaced to connect the end users. The application of PICs is not limited to the telecommunication market. They find application in recognition and sensing tools, optical signal processing, biophotonics, high-speed computing, sources of coherent and incoherent light, etc. In the development of integrated optoelectronic devices, various technological platforms are used: Si, InP, LiNiO_3_, GaAs, GaN, SiO_2_/SiN_x_, polymer photonics, etc. Indium phosphide is one of the basic materials that make it possible to create both active and passive elements [1,2,3,4].

The manufacturing process for InP PIC often requires several sequential etching stages. Etching operations can be carried out using both wet and plasma chemical methods. The advantage of wet etching methods is the ability to form a smooth surface with a minimum level of defects. This provides a low level of optical loss. However, the control of the etching profile in this method is limited [5]. In the fabrication of optical waveguide structures, it is often required to form elements with a high anisotropy (waveguides, gratings, splitters, etc.) while maintaining a smooth surface morphology [6,7,8,9,10]. The methods of plasma chemical etching used for such structures include reactive ion etching (RIE), reactive ion etching in inductively coupled plasma (ICP), electron cyclotron resonance (ECR), and chemically assisted ion beam etching (CAIBE) [6,9,10,11,12,13]. One of the most widely used methods is reactive ion etching in inductively coupled plasma. The advantage of this method is the ability to have independent control of the density and energy of plasma ions. This makes it possible to flexibly control the etching modes.

For the plasma chemical methods of InP etching, the most widespread are processes using gas mixtures based on CH_4_/H_2_ and Cl_2_ [6,7,8,9,10,11,12,13,14,15,16,17,18,19,20,21,22,23,24,25]. Etching in CH_4_/H_2_ gas mixture allows obtaining a smooth surface morphology after etching, with a high aspect ratio of the structures formed. At the same time, the disadvantage of these processes is a usually low etching rate (about 0.1 μm/min or less) due to the formation and redeposition of the passivating polymer [6,15,23,24]. As a result, there is a need for frequent cleaning of the reactor of the equipment from the polymer redeposited on its walls. To reduce the growth of the polymer, the additives Cl_2_ and Ar are introduced into the gas mixture [15,20,21,22,25]. Cyclic etching is carried out when etching stages in CH_4_/H_2_ plasma alternate with cleaning stages in oxygen plasma [15]. Another aspect of this group of processes is that the penetration of hydrogen into the substrate material can lead to the so-called hydrogen passivation. That in consequence can cause the time-dependent instability and degradation of the electrical parameters of manufactured devices [6,26].

Chlorine processes are another widely used subset of plasma chemical processes for InP etching. Etching in pure Cl_2_ plasma can provide high etching rates, in the order of several microns per minute [8,11]. However, such a process does not have sufficient anisotropy. Passivating additives N_2_, O_2_, CH_4_, etc. are introduced into the gas mixture to obtain a profile of structures with a high aspect ratio [6,7,8,13,14,15,16,20,21,22]. However, chlorine processes also have their inherent disadvantages. During the etching process, InCl_x_ components are formed in the reaction products. They have a low-volatility at room temperature. Redeposition of InCl_x_ onto a substrate can lead to the appearance of the so-called micro-masking effect, wherein redeposited InCl_x_ molecules form clusters on the substrate surface that prevent etching. This leads to the formation of a high level of roughness of the substrate surface during the etching process. To prevent this problem, the substrate is usually preheated to 150–200 °C and above before etching [6,7,8,13,14,15,20]. This limits the use of chlorine processes.

A number of studies have been published in which the authors use solutions that make it possible to perform the etching without preheating of the substrate. In [16], ICP etching of InP in a Cl_2_/N_2_ (2:13) gas mixture was carried out at low RF power of 25 W to ensure a smooth substrate surface, and the ratio of Cl_2_ and N_2_ fluxes was tuned to ensure a vertical profile of the elements. In another study [21], ICP etching of InP was carried out in a Cl_2_/CH_4_/Ar gas mixture; the formation of a vertical sidewall and a smooth surface was ensured by matching a set of process parameters: flow ratio between Cl_2_ and CH_4_, ICP and RF powers, and sample holder temperature. The same approach was applied in [22], but other gas mixtures (Cl_2_/N_2_ and Cl_2_/CH_4_/H_2_) were used for etching. In all these studies, the emphasis is on the tuning of plasma parameters that ensure the simultaneous achievement of the required level of anisotropy of the process and the formation of a smooth surface morphology after etching. In our research, the emphasis on the tuning of plasma parameters was shifted to ensure only a high level of anisotropy. Moreover, the suppression of the growth of roughness was ensured by the use of a cyclic etching method.

The aim of this research was to develop a method for ICP etching of a p-i-n InP/InGaAsP heterostructure in Cl_2_-based plasma, which does not require preliminary heating of the substrate. The developed method should be applicable for the manufacturing of integrated optics devices. Accordingly, the etching process should provide a high level of anisotropy and a low level of surface roughness after etching, with a total etching depth up to several microns. Three approaches were applied to the performance of the etching process. In the first case, the etching at the required depth was carried out in one stage. Here, the etching mode was preliminarily selected as providing a high level of anisotropy, but in the process of etching there was a continuous growth of the defect layer thickness; this was expected. When there is no preliminary heating, this approach requires accurate matching of a set of process parameters [16,21,22]. In the second approach, the plasma burning mode remained the same, but the etching process was divided into two stages. At the beginning, as in the first approach, the main stage of etching was carried out to the required depth (including the thickness of the defect layer). Then, at the second stage, after cooling the sample to room temperature, the defect layer was polished in the same plasma burning mode. The polishing principle is the strong dependence of the etching rate of InP/InGaAsP on temperature. The loose defect layer is heated by plasma faster than the bulk material of the substrate, so its removal is also faster. The third approach was a further evolution of the etching process. At the beginning, the plasma burning mode was also left unchanged. The main stage of etching was split into a series of etching cycles with a fixed durability, and with the sample cooled to room temperature after each cycle. The time limit of the etching cycle allowed the thickness of the defect layer to be controlled throughout the etching process. The defect layer formed by the end of each etching cycle was removed at the beginning of the next cycle. Further, in this approach, the parameters of the etching process were optimized. Finally, using the developed process, samples containing a set of integrated waveguide tests were fabricated and their optical losses were measured.

## 2. Materials and Methods

A p-i-n InP/InGaAsP heterostructure was formed on 2-inch semi-insulating InP substrates. The p-InP and n-InP regions were formed with a variable doping profile. The p-InP region was doped with Zn with a maximum doping level of 1 × 10^18^ cm^−3^ and the n-InP region was Si doped with a maximum doping level of 2 × 10^18^ cm^−3^. MQW was formed in the i-region of the p-i-n structure. MQW was based on an InP/InGaAsP superlattice with a total thickness of 400 nm. The total thickness of the epitaxial structure was about 3.5 µm (excluding the semi-insulating buffer layer).

The etching of the heterostructure layers was carried out through a single layer SiN_x_ mask. The SiN_x_ film of 0.8 μm thickness was deposited onto the surface of the wafers by the PECVD method. The topology elements in the dielectric film were formed by RIE in SF_6_-based plasma using a single layer photoresist mask. The waveguide elements were formed using the method of projection i-line lithography.

The elements of the optical waveguides were formed by ICP etching on a Corial 200IL (Corial S.A.S, Meylan, France) system using a Cl_2_/Ar/N_2_ (10/20/20 sccm) gas mixture. In the etching recipe, the ICP power and the pressure during process values were fixed at 400 W and 15 mTorr, respectively. RF power varied in the range of 60–150 W. Before each etching process, the holder with the sample was cooled to room temperature (T = 21 °C). The etching depth and surface condition of the sample were monitored during the etching process using a Horiba LEM-G50 interferometer.

The surface treatment of the samples before etching was carried out in a water solution of 37% HCl in a 1:10 ratio for 1 min, followed by rinsing in deionized water for 1 min and drying in a nitrogen flow. Treatment between etching cycles and after etching was carried out in one of three variants: in deionized water for 2 min, followed by drying in a nitrogen flow and in water solutions of 37% HCl in ratios of 1:5 or 1:3, followed by rinsing in deionized water for 1 min and drying in a nitrogen flow.

The method of scanning electron microscopy was used to obtain microscopic images and measurements of the geometric dimensions of elements. After etching the following measurements were carried out for each sample: the etching depth was assessed by the height of the open region h_Open_, the thickness of the defect layer h_Grass_, the angle of inclination of the sidewalls φ, and the residual thickness of the dielectric mask d_SiN_. The total etching depth h_Total_ was calculated as the sum of the etching depth over the open region and the defect layer thickness: h_Total_ = h_Open_ + h_Grass_. In cycle etching, the etching depth per cycle h_Cycle_ was calculated as the total etching depth h_Total_ divided by the number of etching cycles. The selectivity of the etching process of the heterostructure with respect to the dielectric mask was calculated both over the open region h_Open_ and over the total depth h_Total_ and denoted as S_Open_ and S_Total_, respectively. To determine the selectivity, the etching depth of the dielectric mask h_SiN_ was also calculated.

Then, using the developed ICP etching process, samples containing a set of integrated waveguide tests were fabricated. The tests included waveguides with a width of 1.5 to 2.5 µm, and several Y- and MMI-splitters of different designs. When fabricating the waveguide structures, the upper p-InP layers of the heterostructure were not removed. The separation of the substrate into individual crystals was performed by the cleaving method. An anti-reflective coating was not deposited on the front edges of the crystals. After fabrication, the optical loss in the test elements was measured on an optical stand. A PPCL550 (Pure Photonics LLC, San Jose, CA, USA) tunable semiconductor laser was used as a light source, emitting in the 1530–1560 nm range; a PM20CH (Thorlabs Inc., Newton, MA, USA) optical power meter was used to control the output optical power. Optical radiation input and output to the test elements was handled using tapered SMF28 fiber. 

## 3. Results and Discussions

**Figure 1:**
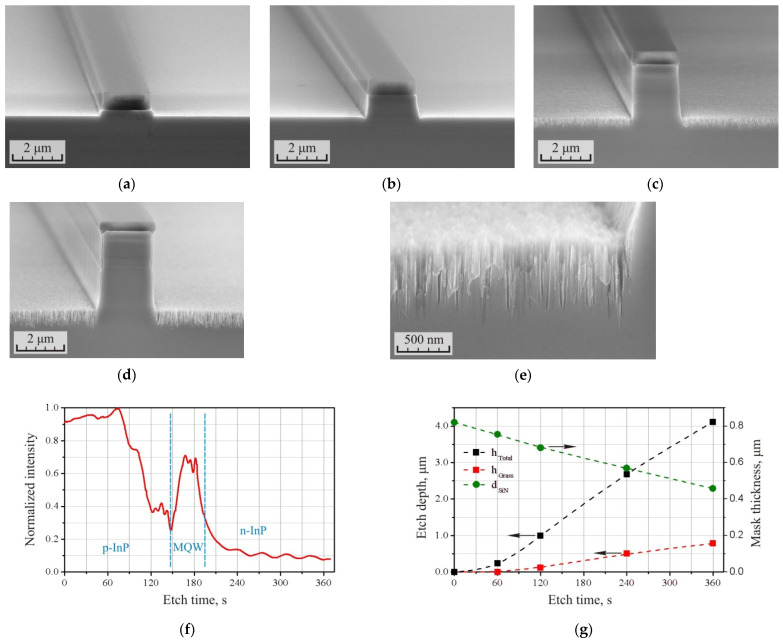
SEM images of the cross-section (**a**–**d**) of the p-i-n InP/InGaAsP structures formed after ICP etching in the Cl_2_/Ar/N_2_ (10/20/20 sccm) gas mixture at the RF power 90 W for 1, 2, 4, and 6 min, respectively, and (**e**) of the sample surface after etching for 6 min. (**f**) The interferogram of the etching process of the p-i-n InP/InGaAsP heterostructure. (**g**) Dependencies of the total etching depth of heterostructure h_Total_, the thickness of the defect layer h_Grass_, and the residual thickness of the SiN_x_ mask d_SiN_ on the etching time.

The results of ICP etching of the p-i-n InP/InGaAsP heterostructure in a Cl_2_/Ar/N_2_ (10/20/20 sccm) gas mixture at a pressure of 15 mTorr and ICP and RF powers of 400 W and 90 W, respectively, are presented in Figure 1 and Table 1. Etching time was 6 min. This etching mode was preliminarily selected as providing high etching anisotropy with a controlled defects growth rate. Figure 1a–d show the cross-sections of waveguides formed after 1, 2, 4, and 6 min of etching, respectively (the SiN_x_ mask was not removed after etching the heterostructure). It could be seen that etching defects on the substrate surface were practically absent after 2 min of etching, while the etching depth of the heterostructure did not exceed 1 μm. The further increase of the etching time resulted in an active growth of the defect layer. In this case, the incline of the waveguide sidewalls also increased, reaching 88.3° after 6 min of etching. 

The appearance of a defect layer after 6 min of etching is shown in Figure 1e. In the used etching mode, they were loose grass-like structures with a high aspect ratio. The thickness of the defect layer reached 1/5 out of the total etching depth (h_Total_ = 4.12 μm). It can also be observed from the figures that defects were formed only on the horizontal surface, and did not appear on the vertical walls of the waveguides. This was ensured by a sufficient level of RF power, which forms the directed motion of plasma ions during the etching process. During etching, a sufficiently strong passivating film is formed on the lateral surfaces of the etched structures as a result of the binding of nitrogen and atoms of the heterostructure, as well as redeposited InCl_x_ from the reaction products. The passivation film cannot be destroyed by the ions of chlorine and argon directed along a tangential trajectory. 

Figure 1f shows an interferogram of the etching process of the p-i-n heterostructure. Here, one could observe the p-InP:MQW and MQW:n-InP transitions which are specific to the heterostructure used. The interferogram also shows a sharp decrease in intensity after 70–75 s of etching, which corresponds to the beginning of the growth of a layer of grass-like defects. In this case, the dependence of the intensity on the thickness of the defect layer was clearly expressed at the initial stages of its growth. After 2 min, the etching intensity decreased to 40% of the maximum, and the thickness of the defect layer was only 0.12 μm. After 4 min, the etching intensity decreased to 13%, and the thickness of the defect layer increased to 0.51 μm. Further, the intensity on the interferogram changed slightly, but the growth of the thickness of the defect layer did not stop, which can be observed in Figure 1g. In the absence of a peak in the MQW region, an exponential dependence can be observed in the interferogram. This shows that the interferogram could be the most informative for controlling the thickness of the defect layer at the initial stages of its formation.

Figure 1g shows the dependences of the total etching depth h_Total_, the thickness of the defect layer h_Grass_, and the residual thickness of the SiN_x_ mask d_SiN_ as a function of the etching time. It can be seen from the dependences that the etching rate of the heterostructure was not linear. At the initial stage, the etching rate was low, but after 1 min the etching rate increased. Then, after 1.5–2 min, the etching rate stabilized, and then in the range of 2–6 min, the dependence became quasi-linear. The tendency to limit the growth of the defect layer in the range up to 6 min was either not observed or was weakly expressed. This means that with a further increase in the etching time, the thickness of this layer would continue to grow. During other experiments, the depth of the defect layer reached 2 μm, which was also not a limit. In this case, it can be observed on the graph that the residual thickness of the dielectric mask decreased linearly throughout the etching stage without a noticeable acceleration or inhibition.

Table 1 shows the dependences of the process selectivity for the open area S_Open_ and for the total etching depth S_Total_. Due to the nonlinearity of the etching rate of the heterostructure at the initial stage, the selectivity of the process was 3.6, which in quite low, but with time the average selectivity of the etching process began to increase, reaching 9.2 after 6 min in the open region and 11.4 considering the depth of the defect layer.

The presented dependencies could be explained as follows. At the initial stage of etching, the sample temperature is close to room temperature (T = 21 °C). A sufficiently strong passivating film is formed on the opened surfaces of the heterostructure during the interaction of nitrogen ions with the substrate. Redeposition of InCl_x_ also occurs, which enhances surface passivation. The energy of plasma ions is not sufficient to effectively remove the passivating layer and thus the etching rate is relatively low. Due to the low etching rate, the etching surface remains smooth. Further, under the influence of plasma ion bombardment, the sample surface heats up, the balance of the process shifts towards desorption of the passivating film, more and more chlorine and argon ions begin to interact with InP, and the etching rate increases. However, in the process of etching, the concentration of InCl_x_ components, which have a relatively low volatility, increases in the reaction products. Due to the fact that the temperature of the sample is not sufficient, indium chlorides continue to deposit onto the substrate. This leads to the micro-masking effect of the substrate surface. This, in turn, leads to the non-uniform etching of the substrate and the appearance of so-called grass-like defects. The roughness of the substrate surface increases. With a further increase in the substrate temperature to 150–200 °C, indium chlorides from the reaction products should be efficiently desorbed from the substrate surface. Then, the growth of the thickness of the defect layer should either stop or continue in the opposite direction (since, in theory, the etching rate of the loose grass-like layer should be higher than that of the bulk material). However, in our case, this did not happen. The stabilization of the etching rate of the heterostructure was observed already after 1.5–2 min of etching, and the increase in the thickness of the grass-like layer was just beginning here. Accordingly, the heating of the samples by the plasma was insufficient for the effective desorption of the InCl_x_ components from the reaction products, and the micro-masking of the substrate surface continued.

It would seem obvious that the presented etching mode is only limitedly applicable for the formation of optical waveguides. Its advantages include high anisotropy with the formation of the smooth lateral surfaces of waveguides and a good selectivity with respect to the SiN_x_ mask. However, the defect layer formed during etching does not allow for the creation of any complex optical devices that require ohmic contacts to the lower layers of the heterostructure and/or passive waveguides, when it is necessary to etch the upper p-layers of the heterostructure. 

**Figure 2:**
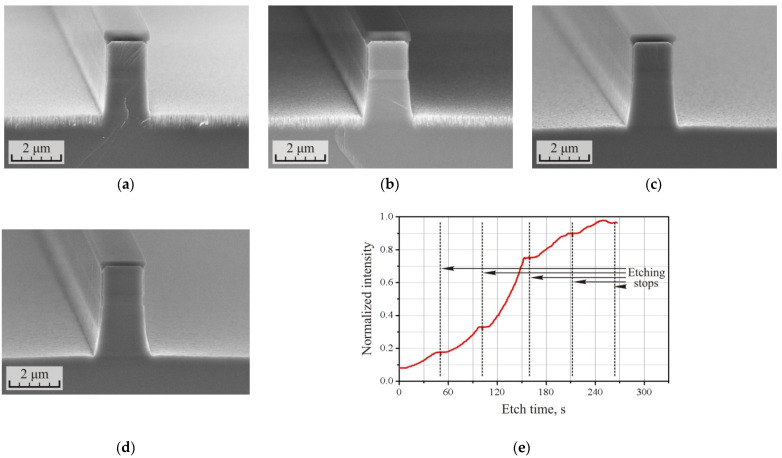
(**a**–**d**) SEM images of the cross-section of the p-i-n InP/InGaAsP structures formed after ICP etching in the Cl_2_/Ar/N_2_ (10/20/20 sccm) gas mixture at the RF power 90 W before and after 1, 3, and 5 after-etching polishing cycles, respectively. (**e**) The interferogram of the polishing process of the sample surface.

However, as noted earlier, at the initial stage of etching, when the etching rate is relatively low, etching proceeds without the formation of a defect layer. The etched surface of the heterostructure remains smooth. There was also an assumption that the loose grass-like defect layer in the plasma should be heated faster than the bulk material of the substrate, and, accordingly, the rate of its etching at the initial stage should be higher. These ideas were tested in the following experiment, the results of which are presented in Figure 2 and Table 2. 

The etching mode remained the same, while etching time was reduced to 5 min. The total etching depth h_Total_ was 3.64 microns with a defect layer thickness of 0.64 µm. The cross-section of the p-i-n structure after etching is shown in Figure 2a. Then, the sample was cleaved into parts, each of which was subjected to one to five short etching cycles in the same mode, but the duration of each cycle was limited in time to 40–45 s. Between etching cycles, the holder with the samples was unloaded from the chamber and cooled to room temperature. Cross-sections of p-i-n structures after 1, 3, and 5 short-term etching cycles are shown in Figure 2b–d. As can be seen from the figures and Table 2, the thickness of the defect layer on the substrate surface decreased with an increase in the number of etching cycles. After five etching cycles, the defect layer was barely visible, and its thickness was within 30 nm. The application of short etching cycles ensured the polishing of the sample surface. 

Changes in surface condition are reflected in the etching interferogram shown in Figure 2e. After each cycle of polishing etching, the intensity of the signal from the interferometer increased. Moreover, if in the first two cycles of polishing, the growth rate was relatively low, this was associated with the large thickness of the grass-like layer. Then, during the third polishing cycle, the signal intensity changed dramatically. During the further cycle of polishing, the intensity growth rate slowed down abruptly, and at the end of the last cycle it even sagged slightly. This indicated the almost complete depletion of the defect layer. Here, the interferometer acts as a powerful tool for monitoring the surface condition during etching and the subsequent polishing.

An interesting and unexpected feature of the developed process was the constancy of the total etching depth h_Total_ of the heterostructure during the after-etching polishing cycles. The assumption that a loose grass-like defect layer would heat up faster during etching in plasma and, accordingly, would be etched faster turned out to be correct. However, the experimental results were even better. The limitation of the polishing cycle time to 40–45 s meant that the bulk material of the substrate did not have time to warm up enough and its etching did not start.

Despite the fact that the etching process has become much more attractive for the fabrication of integrated optics devices, a number of disadvantages can be highlighted. Firstly, at the initial etching stage, an uncontrolled increase in the thickness of the defect layer was observed. The greater the etching depth required to form the device, the greater the depth of the defect layer formed, and thus more polishing cycles would be required. This challenge is critical when a precision etching depth is required.

Secondly, as could be seen from Figure 2a–d, before polishing the waveguide had an almost vertical profile. After the complete removal of the defect layer, the waveguide profile acquired a noticeable bend radius; its width in the upper plane differed significantly from its width at the base. Apparently, this is also associated with the plasma heating of the sample. The heating of bulk material at the base of the waveguide is not sufficient for etching it, while in the upper plane of the waveguide the material warms up when etching begins. The effect does not have time to completely manifest itself and, certainly, a decrease in the polishing cycle time could improve the situation. However, at the same time, this would require an increase in the number of polishing cycles until the thickness of the defect layer is depleted. It is also obvious that at lower etching depths, the effect of process anisotropy reduction would be less pronounced. On the one hand, the waveguide would stand out less over the surface of the substrate; accordingly, the temperature gradient would be less pronounced. On the other hand, the thickness of the defect layer would also be smaller, which means that fewer polishing cycles would be required.

Thirdly, a decrease in the overall selectivity of the process with an increase in the number of polishing cycles should be noted, which can be observed in the corresponding columns of Table 2. This challenge reduces the maximum achievable etching depth of the heterostructure and requires a thicker etching mask. This is due to the fact that etching of the heterostructure does not start immediately; even for etching a loose defect layer, it takes time until the optimum temperature is reached. At the same time, the etching rate of SiN_x_ is practically temperature independent, and etching starts immediately after the plasma is ignited. This could be evidenced by a decrease in the selectivity parameter of the process in the open region S_Open_. The removal of the defect layer was not fast enough in comparison with the decrease in the thickness of the dielectric mask. In this case, reducing the polishing cycle time and increasing the number of cycles would only exacerbate the situation. 

After analyzing the obtained results, we decided to change the approach to solving the problem of etching. The introduction of polishing cycles undoubtedly improved the results and should be used in the development of the etching process. However, during the etching process, it is necessary to constantly monitor the thickness of the defect layer, not allowing it to go beyond the delineated boundary. This boundary was determined to be in the range of 0.1–0.2 µm to achieve the accuracy required in our work. In the updated solution, instead of one main etching stage followed by subsequent after-etching polishing cycles, the etching process was split into a few cycles with polishing at the beginning of each etching cycle. 

**Figure 3:**
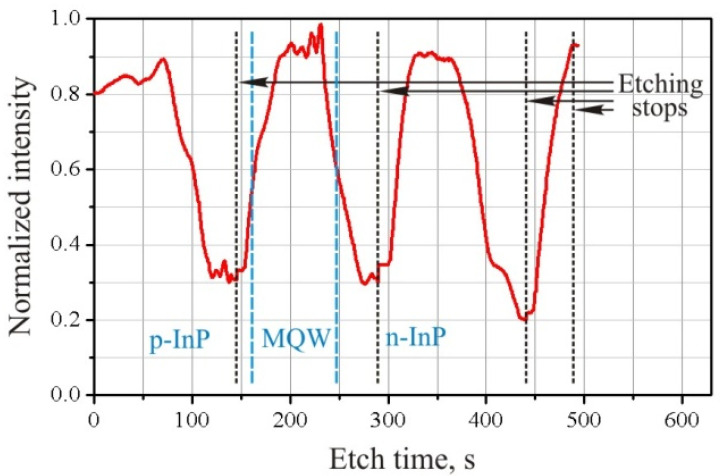
The interferogram of the ICP etching process of the p-i-n InP/InGaAsP heterostructure in the Cl_2_/Ar/N_2_ (10/20/20 sccm) gas mixture at the RF power 90 W with the division of the process into three cycles for 2 min 20 s and finish polishing for 40 s.

An interferogram of the updated etching process is shown in Figure 3. The plasma burning mode was left unchanged, as in the two previous experiments. However, the main etching stage was divided into three cycles for 2 min 20 s. At the end, the final polishing of the surface was carried out in the same mode for 40 s. After each etching cycle, the sample holder was unloaded from the chamber and cooled to room temperature. As can be seen from the interferogram, the removal of the defect layer was carried out starting from the second cycle, at the beginning of etching, when the sample temperature was still insufficient for etching the bulk material. This is reflected in the increase in signal intensity from the interferometer. Further, a shelf was observed in the interferogram; at this stage of the cycle, the etching rate of the heterostructure was relatively low and the thickness of the defect layer did not grow. At the third stage of the cycle, the temperature reached a critical value, the etching rate of the heterostructure increased, and an active increase in the thickness of the defect layer began. Here, the duration of the etching cycle was selected based on two criteria. First, at the end of the etching cycle, the thickness of the defect layer should not exceed 0.1–0.2 µm. Second, the thickness of the defect layer had to be such that at the beginning of each subsequent etching cycle, it would be completely removed. In this case, the etching process would be controlled until the thickness of the dielectric mask would be completely depleted. It can also be seen from the interferogram that changing the etching mode still makes it possible to identify the transitions between the p-InP:MQW and MQW:n-InP regions, and thus to bind to the current etching depth. 

**Figure 4:**
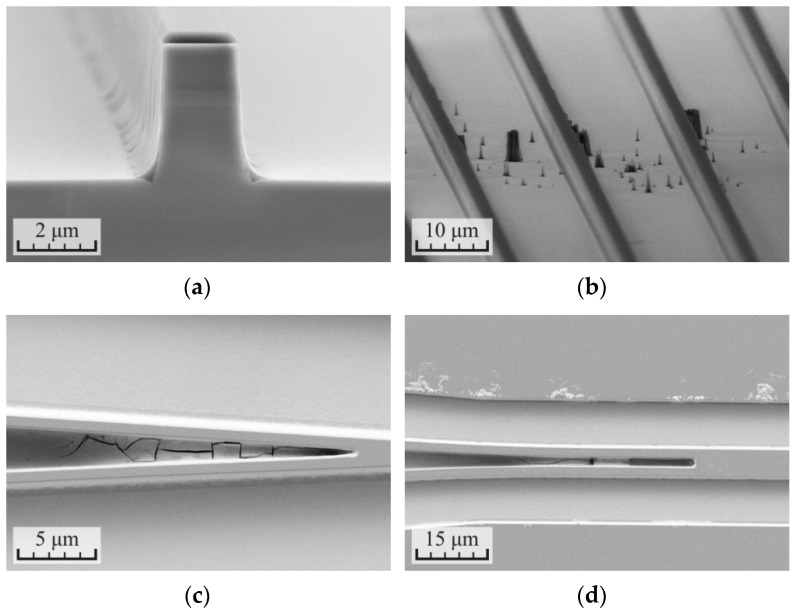
SEM images of the p-i-n InP/InGaAsP structures formed after ICP etching in the Cl_2_/Ar/N_2_ (10/20/20 sccm) gas mixture at the RF power 90 W with the division of the process into three cycles for 2 min 20 s and finish polishing for 40 s: (**a**) cross-section of the waveguide, (**b**) side view of the waveguides, (**c**) Y-splitter, and (**d**) MMI-splitter.

However, during the control of samples after etching on SEM, etching defects were found. Microscopic images of the elements of the waveguide structures are shown in Figure 4. The defects were concentrated in specific sites: on the inner corners of the waveguides, Figure 4a; on the dielectric mask defects, Figure 4b; and between closely located waveguides and on the surface of the dielectric mask, Figure 4c,d.

It was suggested that defects are formed due to redeposition of InCl_x_ from the reaction products to the surface of the samples. The introduction of relatively short cycles of etching reduced the average temperature of the samples during the etching process and could aggravate the situation. Nevertheless, the cause of such segregation of the defects on the surface of the samples was unclear. The situation cleared when the samples were controlled into an optical microscope immediately after etching. On the surface of the samples, within a few minutes after unloading from the vacuum chamber, the growth of the defects on all elements of the relief were observed. This was due to the fact that InCl_x_ salts, having high hygroscopicity, began to actively take moisture from the surrounding atmosphere. As a result, the droplets of the salt solution were formed on the surface of the samples. Subsequently, when the samples were loaded into the SEM vacuum chamber, the moisture quickly evaporated and the salts collected from the entire surface of the samples were concentrated on the elements of the relief to which the drops of the solution adhered. The surface of the samples was again saturated with moisture after unloading from the vacuum chamber. 

To solve this problem, it was decided thar after each etching cycle wet treatment of the samples would be carried out in order to rinse off the etching reaction products from the surface. Since contaminations on the surface showed high hygroscopicity, they should be well soluble in water. One of the treatment variants was rinsing in deionized water for 2 min, followed by drying in the flow of nitrogen. In another variant, it was proposed to additionally polish the sample surface in the solution of the polishing etcher. From the literature, it is known that the water solutions of HCl can vastly change the etching rate of InP from nanometer units to tens of microns per minute depending on the acid concentration. In our experiments, two variants of dilution of 37%HCl in deionized water 1:5 and 1:3 were selected. Higher concentrations were not used since in the 1:2 dilution the etching rate of InP was already submicron per minute, which could change the etching profile of the heterostructure.

**Figure 5:**
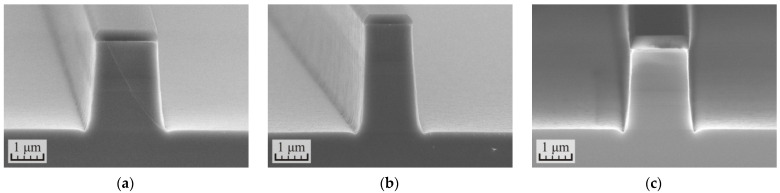
SEM images of the cross-section of the p-i-n InP/InGaAsP structures formed in the cyclic ICP etching process in the Cl_2_/Ar/N_2_ (10/20/20 sccm) gas mixture at the RF power 90 W with different treatments between etching cycles: (**a**) in a deionized water for 2 min, (**b**) in a water solution of 37%HCl (1:5) for 1 min, and (**c**) in a water solution of 37%HCl (1:3) for 1 min.

The results of the cyclic etching of the samples with wet treatments between etching cycles are shown in Figure 5. In all three cases, the reaction products from the surface of the samples were effectively removed and the defects shown in Figure 4 were not observed. At the same time, there was no advantage in treating samples in water solutions of HCl over rinsing in deionized water. Apparently, the polishing effect of the plasma dominated here. Therefore, in subsequent experiments, the treatment of samples between etching cycles was carried out in deionized water for simplicity.

**Figure 6:**
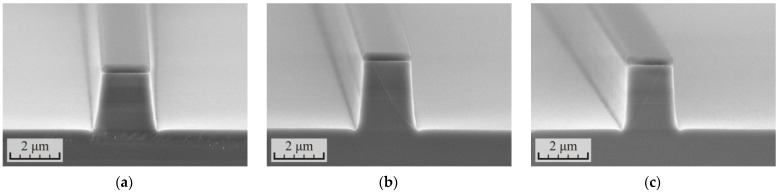
SEM images of the cross-section of the p-i-n InP/InGaAsP structures formed in the cyclic ICP etching process in the Cl_2_/Ar/N_2_ (10/20/20 sccm) gas mixture at the RF power 90 W with different time durations and numbers of etching cycles: (**a**) 4 cycles for 90 s with finish polishing for 25 s, (**b**) 3 cycles for 120 s with finish polishing for 40 s, and (**c**) 2 cycles for 150 s with finish polishing for 40 s.

Then, a series of experiments was carried out with the aim of increasing the anisotropy of the etching process and increasing its selectivity with respect to the SiN_x_ dielectric mask. In the first experiment, the influence of the etching cycle time was studied. The results are presented in Figure 6 and Table 3. The number and time of etching cycles were chosen so as to have comparable etching depths. From the data presented, a relationship can clearly be observed between the etching cycle duration and the angle of inclination of the waveguide sidewalls. With a cycle time of 90 s, the tilt angle was 82.1°, while at 120 s, the angle increased to 85.6°, and upon reaching the cycle time of 150 s, the tilt angle increased to 87.7°. The increase in the angle is probably due to the fact that, at the beginning of the etching process, when the sample is too cold, etching of the heterostructure does not occur. As the sample is heated by the plasma, the etching begins on surfaces that are perpendicular to the flow of plasma ions. At the same time, the energy of the ions arriving at the sidewalls of the waveguides at tangential angles is insufficient to overcome the forming passivation layer. At this stage, the width of the waveguide grows at the base. However, as the sample warms up further, the lower ion energy is required to start the etching process, and the growth of the waveguide width at the base stops. This contributes to an increase in the verticality of the waveguide side walls with longer etching cycle duration and other constant process parameters.

With an increase in the cycle time, the proportion of time increases when the etching of the heterostructure has occurred in the cycle; therefore, the process selectivity also increases. At the cycle time of 90 s, the selectivity with respect to the total etching depth S_Total_ was 5.4, while upon reaching the cycle time of 150 s, the selectivity increased to 6.9.

The time of the finish polishing of the samples was chosen so as to minimize the thickness of the defect layer. In all three cases, the thickness of the defect layer did not exceed 30 nm after polishing. However, with an increase in the cycle time, the thickness of the defect layer formed by the end of the etching cycle also increased. With the etching cycle time of 90 s, one polishing for 25 s was sufficient to remove the defect layer, while with an increase in the cycle time to 120 s, the finish polishing time increased to 40 s. Then, when the etching cycle time reached 150 s, the polishing had to be carried out in two cycles: 40 + 20 s. 

In principle, to obtain more vertical waveguide sidewalls, the etching cycle time can be increased further. However, here it should be considered that at the cycle time of 150 s, the process already becomes non-stationary. The defect layer at the beginning of each cycle does not have time to be completely removed, and with each subsequent etching cycle, its thickness will increase. If a large etching depth is required for the manufacture of a device and a high precision in depth is not required, then this will not be as critical.

**Figure 7:**
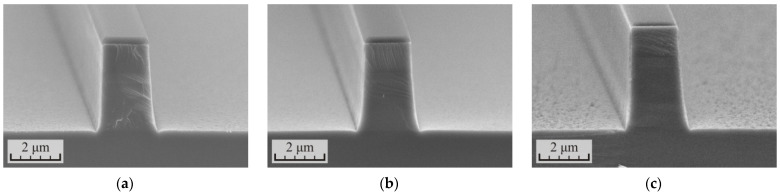
SEM images of the cross-section of the p-i-n InP/InGaAsP structures formed in the cyclic ICP etching process in the Cl_2_/Ar/N_2_ (10/20/20 sccm) gas mixture at different levels of the RF power: (**a**) 90 W, (**b**) 120 W, and (**c**) 150 W.

In the second experiment, the influence of the increasing RF power was studied. An increase in RF power leads to an increase in the bias potential on the surface of the etched sample and, accordingly, to a more directional movement of plasma ions. It was assumed that this solution would increase the anisotropy of the etching process. In this case, the increase in RF power should be limited, since this increases the energy of the ions bombarding the substrate and this can lead to an accelerated growth in the thickness of the defect layer. The samples were also etched at about the same depth for an accurate comparison of the modes with each other. However, the total etching depth was increased to 3.5–4 μm. The heterostructure was etched in three cycles and the cycle time in each case was chosen such that the sample was etched by 1.2–1.3 μm per cycle. The results of the experiment are presented in Figure 7 and Table 4.

As it turned out, at a fixed value of the etching depth per cycle, the angle of inclination of the waveguide sidewalls practically did not change with increasing RF power. The angle varied in the range of 86.7–87.3°. Even a slight decrease was observed with increasing RF power. This could be due to a slight decrease in the selectivity of the etching process. At the same time, if we compare the data from Table 3 and Table 4 for samples with an etching time of 3 × 120 s, an increase in RF power from 90 to 120 W increased the angle of inclination of the waveguide sidewalls from 85.6 to 86.8°, while the depth of sample etching per cycle increased from 0.92 to 1.18 μm. Apparently, here the decisive role in increasing the anisotropy of the process was performed not by the increase of the RF power itself, but the increase of the etching depth per cycle. 

As expected, an increase of the RF power could not affect the growth of the defect layer. After increasing the RF power to 120 W, the sample was also polished in one stage with a duration of 50 s. The process used has a safety margin. However, at 150 W, even three cycles of polishing for 40 s were not enough to completely remove the defect layer. In addition, it was necessary to reduce the RF power to 120 W to carry out the polishing. However, even after this, the residual roughness on the sample surface was about 150 nm. This residual roughness was difficult to remove further. Apparently, the reason was that the elements of the residual roughness had a low aspect ratio. Heating the sample surface in plasma did not create a sufficient temperature gradient between the residual relief elements and the bulk material of the sample. Therefore, the smoothing of the residual relief proceeded slowly.

**Figure 8:**
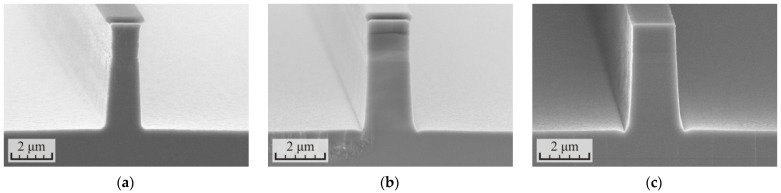
SEM images of the cross-section of the p-i-n InP/InGaAsP structures formed in the cyclic ICP etching process in the Cl_2_/Ar/N_2_ (10/20/20 sccm) gas mixture at different levels of the RF power: (**a**) 60 W, (**b**) 75 W, and (**c**) 90 W.

The previous two experiments showed an improvement in the anisotropy of the process with an increase in the etching depth per cycle (h_Cycle_). Based on this, it was decided to increase this value as much as possible. To do this, it was necessary to slow down the growth of the defect layer during the etching process. This was achieved by reducing the RF power to 60 and 75 W, which led to a decrease in plasma ion energy. The third etching mode with the RF power of 90 W was added for comparison with the results of previous experiments. Furthermore, the total etching depth was again increased to 4.4–5.2 µm. The results of the experiment are presented in Figure 8 and Table 5.

Comparing the etching results at RF power 90 W from Table 4 and Table 5, it can be noted that an increase in the etching depth per cycle from 1.18 to 1.45 µm allowed the angle of inclination of the waveguide sidewalls to further increase from 87.3 to 88.2°. At the same time, the selectivity of the process practically reached the limit. The sample surface remained smooth after plasma polishing and the roughness did not exceed 30 nm. However, in the second process, two polishing stages were required instead of one. 

Reading the RF power to 75 W allowed increasing the cycle time from 160 to 230 s. Although the count of etching cycles decreased from 3 to 2, the final etching depth turned out to be somewhat increased. These changes allowed for the further increase of the angle of inclination of the waveguide sidewalls to 88.8°. This result again confirms that, with an increase in the parameter of the etching depth per cycle, the anisotropy of the process improves. Here, the radius of bending at the base of the waveguide noticeably decreased. A good addition was a significant increase in the selectivity of the etching process from 7.2 to 9.0; one stage with a duration of 50 s was enough to polish the surface.

Further, reducing the RF power to 60 W still increased the selectivity of the process to 9.9. However, the etching profile of the waveguide acquired a noticeable bending in the shape of a sandglass. The anisotropy of the process decreased due to an increase in the lateral etching of the waveguides. A particularly noticeable lateral etching was observed in the MQW region consisting of alternating InP/InGaAsP layers. The roughness of the waveguide sidewalls also increased. In this case, the plasma ion energy is no longer enough, and they acquire a large spread of the movement vectors. The result of the process could possibly be improved by optimizing other plasma parameters (gas mixture, pressure, ICP power, etc.). 

**Figure 9:**
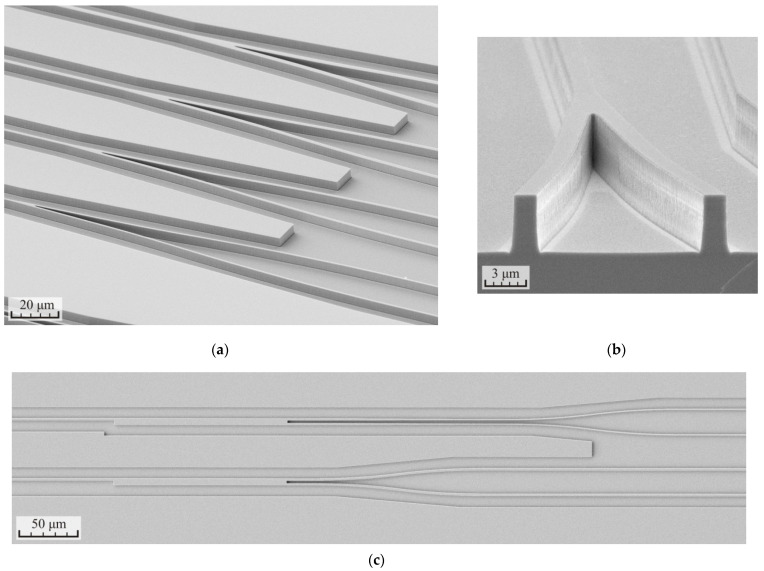
SEM images of the test waveguide structures formed in the p-i-n InP/InGaAsP heterostructure: (**a**) side view of the Y-splitters, (**b**) cross section of the Y-splitter, and (**c**) top view of the MMI-splitters.

In the final part of the work, the samples were fabricated with different test structures: waveguides with a width of 1.5 to 2.5 μm and several designs of Y- and MMI-splitters. SEM images of some fabricated elements are shown in Figure 9. The mode number three from Table 5 was used for the formation of the elements. The main stage of etching included three cycles for 160 s and the polishing was carried out by two cycles for 40 s. The total depth of etching was 4.3–4.4 μm, the surface roughness did not exceed 30 nm, and the angle of inclination of the waveguide sidewalls was 88.2°. The selectivity to the SiN_x_ dielectric mask was equal to 7.2. The residual layer of the dielectric mask was removed from the surface of the waveguides after etching the heterostructure.

The measurement results of the optical loss of some test elements are presented in Table 6. 

In the process of fabricating waveguides, the removal of the upper high doped p-InP layers of the heterostructure was not carried out. This explains the relatively high propagation loss in waveguides. Usually, in these high doped p-layers, the main absorption of the optical wave occurs. In [27], which had a similar configuration of the design and the heterostructure of the integral waveguide with the p-layers, its propagation loss reached 8–10 dB/cm. In practice, both types of waveguides are used in devices. The sections of so-called active waveguides (where p-layers of the p-i-n structure are not removed) are used to actively control the optical wave (refraction index and absorption coefficient of the waveguide are changing). Through ohmic contacts that are formed in these sections, an electric field is applied to the heterostructure. On passive sections where active control does not occur, the upper high doped p-layers of the heterostructure are carried out to minimize optical losses. An additional reason for relatively high losses is that the heterostructure used requires further optimization. 

Furthermore, on the test samples there were several designs of the optical splitters. Table 6 shows the measurement results of insertion loss for the two most successful designs. The insertion loss of the splitters did not exceed 0.8 dB, which can be considered a good level [28,29,30,31,32,33].

## 4. Conclusions

The results of the development of the method for ICP etching of an InP/InGaAsP heterostructure in a Cl_2_/Ar/N_2_ gas mixture were presented in this work. Etching started at room temperature without preliminary heating of the substrate. The initially selected basic etching mode ensured high anisotropy, smoothness of the waveguide sidewalls, and a sufficiently high selectivity with respect to the SiN_x_ mask. However, due to the absence of preheating, insufficient substrate temperature during etching led to the formation of a loose grass-like defect layer on the horizontal surfaces of the heterostructure. Thus, the thickness of the defect layer was 1/5 out of the total etching depth of 4.12 μm. In this case, the thickness of the defect layer was not fixed, but continued to grow as the etching depth increased. This limits the applicability of this etching process for optical applications.

A different approach was proposed in the work. The plasma burning mode during the ICP etching process was left unchanged. However, instead of a single etching process, time-limited etching cycles were introduced, with the samples being cooled between cycles to room temperature. It was shown that this makes it possible not only to effectively control the growth of the thickness of the defect layer, but also to reverse it. After etching, a smooth substrate surface was formed. In comparison with other works on ICP etching of InP in Cl_2_-based plasma, which also did not use preliminary heating of the substrate, the developed etching method makes it possible to more freely control the plasma parameters to achieve the required levels of anisotropy and roughness of the waveguide surface, not focusing primarily on the suppression of the growth of the defect layer.

The additional optimization of the parameters of the basic etching mode made it possible to achieve an angle of inclination of waveguide sidewalls of 88.8° at etching depths more than 4.5 μm. The efficiency of the method for suppressing the growth of the defect layer was demonstrated by the fact that, after etching, the surface roughness of the substrate did not exceed 30 nm, and could be further reduced. In this case, the selectivity of the etching process with respect to the SiN_x_ mask reached 9, which is a sufficient level for most practical applications.

Using the developed etching method, test waveguide structures were fabricated. The propagation loss of active 2 μm wide waveguides was 11 ± 1.5 dB/cm at 1550 nm. The relatively high propagation loss in waveguides could be explained by the fact that, during fabrication, the upper highly doped p-InP layers of the heterostructure were not etched. This also requires further optimization of the used heterostructure. In addition to waveguides, the test structures contained optical splitters. The insertion loss of the developed Y- and MMI-splitters did not exceed 0.8 dB. 

## Figures and Tables

**Table 1:** The results of ICP etching of the p-i-n InP/InGaAsP heterostructure in the Cl_2_/Ar/N_2_ (10/20/20 sccm) gas mixture at the RF power 90 W for 1, 2, 4, and 6 min.

Etch Time, (min)	h_Total_, (μm)	h_Open_, (μm)	h_Grass_, (μm)	h_SiN_, (μm)	φ, °	S_Total_	S_Open_
1	0.23	0.23	0	0.065	-	3.6	3.6
2	1.00	0.88	0.12	0.14	84.0	7.1	6.3
4	2.68	2.17	0.51	0.25	87.3	10.7	8.6
6	4.12	3.33	0.79	0.36	88.3	11.4	9.2

**Table 2:** The results of ICP etching of the p-i-n InP/InGaAsP heterostructure in the Cl_2_/Ar/N_2_ (10/20/20 sccm) gas mixture at the RF power 90 W using after-etching polishing cycles.

Polishing Cycles	h_Total_, (μm)	h_Open_, (μm)	h_Grass_, (μm)	h_SiN_, (μm)	S_Total_	S_Open_
Before	3.64	3.00	0.64	0.34	10.7	8.8
1	3.66	3.10	0.56	0.39	9.4	7.9
3	3.62	3.41	0.21	0.46	7.9	7.4
5	3.64	3.61	0.03	0.58	6.3	6.2

**Table 3:** The results of cyclic ICP etching of the p-i-n InP/InGaAsP heterostructure in the Cl_2_/Ar/N_2_ (10/20/20 sccm) gas mixture at the RF power 90 W with different time durations and numbers of etching cycles.

Etch Time, (s)	h_Total_, (μm)	h_Cycle_, (μm)	h_Open_, (μm)	h_Grass_, (μm)	h_SiN_, (μm)	φ, °	S_Total_	S_Open_
4 × 90 + 25	2.322	0.58	2.30	0.022	0.43	82.1	5.4	5.3
3 × 120 + 40	2.760	0.92	2.73	0.03	0.45	85.6	6.1	6.0
2 × 150 + 40 + 20	2.582	1.29	2.56	0.022	0.37	87.7	6.9	6.8

**Table 4:** The results of cyclic ICP etching of the p-i-n InP/InGaAsP heterostructure in the Cl_2_/Ar/N_2_ (10/20/20 sccm) gas mixture at different levels of the RF power.

RF Power, (W)	Etch Time, s	h_Total_, (μm)	h_Cycle_, (μm)	h_Open_, (μm)	h_Grass_, (μm)	h_SiN_, (μm)	φ, °	S_Total_	S_Open_
90	3 × 140 + 50	3.535	1.18	3.50	0.035	0.50	87.3	7.1	7.0
120	3 × 120 + 50	3.546	1.18	3.52	0.026	0.55	86.8	6.4	6.4
150	3 × 110 + 3 × 40 ^1^	4.050	1.35	3.90	0.150	0.63	86.7	6.4	6.2

^1^ The finish polishing was carried out at the power of 120 W.

**Table 5:** The results of cyclic ICP etching of the p-i-n InP/InGaAsP heterostructure in the Cl_2_/Ar/N_2_ (10/20/20 sccm) gas mixture at different levels of the RF power.

RF Power, (W)	Etch Time, (s)	h_Total_, (μm)	h_Cycle_, (μm)	h_Open_, (μm)	h_Grass_, (μm)	h_SiN_, (μm)	φ, °	S_Total_	S_Open_
60	2 × 360 + 40	5.183	2.59	5.15	0.033	0.53	87.1	9.9	9.8
75	2 × 230 + 50	4.576	2.29	4.55	0.026	0.51	88.8	9.0	8.9
90	3 × 160 + 2 × 40	4.365	1.45	4.34	0.025	0.61	88.2	7.2	7.1

**Table 6:** The results of measurements of the optical loss of the test elements.

Component	Parameter	Value
Waveguide 2 um width	Propagation loss	15 ± 1.5 dB/cm at 1530 nm
		12.9 ± 1.5 dB/cm at 1540 nm
		11 ± 1.5 dB/cm at 1550 nm
		11 ± 1.5 dB/cm at1560 nm
Waveguide 1.5 um width	Propagation loss	14.8 ± 1.5 dB/cm at 1550 nm
Waveguide 2.5 um width	Propagation loss	11 ± 1.5 dB/cm at 1550 nm
MMI-splitter	Waveguide width	2 μm
	Insertion loss	<0.8 dB
Y-splitter	Waveguide width	1.5 μm
	Insertion loss	<0.7 dB
